# Differential expression of mRNA-miRNAs related to intramuscular fat content in the longissimus dorsi in Xinjiang brown cattle

**DOI:** 10.1371/journal.pone.0206757

**Published:** 2018-11-09

**Authors:** Na Li, Yang Zhang, Hai-Peng Li, Ling Han, Xiang-Min Yan, Hong-Bo Li, Wei Du, Jin-Shan Zhang, Qun-Li Yu

**Affiliations:** 1 Department of Food Science and Engineering, Gansu Agricultural University, Lanzhou, Gansu, China; 2 Department of Research Livestock, Xinjiang Academy of Animal Science, Urumqi, Xinjiang, China; 3 Department of Animal Sciences, Chinese Academy of Agricultural Sciences, Beijing, China; Kunming University of Science and Technology, CHINA

## Abstract

In this study, we examined the role of mRNAs and miRNAs in variations in intramuscular fat content in the longissimus dorsi muscle in Xinjiang brown cattle. Two groups of Xinjiang brown cattle with extremely different intramuscular fat content in the longissimus dorsi were selected for combined of miRNA and mRNA analysis using an RNA-Seq. In total, 296 mRNAs and 362 miRNAs were significantly differentially expressed, including 155 newly predicted miRNAs, 275 significantly upregulated genes, 252 significantly upregulated miRNAs, 21 significantly downregulated genes and 110 significantly downregulated miRNAs. The combined miRNA and mRNA analysis identified 96 differentially expressed miRNAs and 27 differentially expressed mRNAs. In all, 47 upregulated miRNAs had a regulatory effect on 14 differentially downregulated target genes, and 49 downregulated miRNAs had a regulatory effect on 13 upregulated target genes. To verify the sequencing results, 10 differentially expressed genes (DEGs) and 10 differentially expressed miRNAs were selected for qRT-PCR. The qRT-PCR results confirmed the sequencing results. The results of this study shed light on the molecular regulation of bovine adipose tissue, which might help with the development of new strategies for improving meat quality and animal productivity in beef cattle to provide healthier meat products for consumers.

## Introduction

Xinjiang brown cattle are an improved breed with an independent intellectual property right. Numbering over 1.7 million, Xinjiang brown cattle are the main cattle breed farmed in Xinjiang. However, no systemic breed selection has been performed since the breed was established [1], leading to large fluctuations in the quality of snow beef and greatly impairing the international competitiveness of Xinjiang brown cattle products. The molecular mechanisms underlying fat deposition in Xinjiang brown cattle have become a popular topic of research.

MicroRNAs (miRNAs) constitute a class of endogenous, small non-coding RNAs of approximately 21–25 nucleotides (nt) in length [2]. In mammals, miRNAs regulate signaling pathways related to fat production by inhibiting transcriptional factors involved in adipogenesis. miRNAs have been reported to negatively regulate fat production by acting on insulin signaling and C/EBPs [3]. In 3T3-L1 cells, miR-320 and miR-29 act directly on phosphatidyl inositol kinase (PIK), which is an important factor in insulin signaling, to reduce insulin activity and inhibit adipocyte formation [4]. miR-27a and miR-27b act on the prohibitin (PHB) gene to negatively regulate adipocyte formation by inhibiting PHB [5].

However, the identification of miRNA target genes remains a considerable challenge because the effective target sequences of miRNAs may not be conserved [6]. In addition, to regulate mRNAs, the bases in the seed sequence of miRNAs bind 6 complementary bases at the target site of the mRNA [7], which increases the complexity of the regulatory effect of miRNAs on mRNAs. The following strategy has been shown to be practically effective: first, small RNA (sRNA) sequencing is performed to identify and predict differentially expressed miRNA target genes; then, the differentially expressed mRNAs are screened; and finally, a correlation analysis between the target genes and differentially expressed genes (DEGs) is conducted to determine the regulatory relationship between the miRNAs and mRNAs. This method has been widely applied to identify miRNA target genes in different tissues [8, 9]. In the present study, sRNA sequencing, prediction of differentially expressed miRNA target genes and screening for differentially expressed mRNAs were performed via RNA-Seq. Then, a correlation analysis of the DEGs was performed to determine the key factors that regulate fat deposition in Xinjiang brown cattle. The study findings provide a theoretical basis for screening effective biomarkers of lipid metabolism.

## Materials and methods

### Animals

The cattle were provided by the Yili Tianxi Breeding Company. Thirty-three healthy castrated Xinjiang brown cattle aged 10–12 months were selected for a 22-month fattening experiment. All animal procedures were approved by the IACUC of Institute of Research Livertock, Xinjiang Academy of Animal Science (approval number: 20150501).

### Slaughtering, segmentation and sampling

**Fig 1:**
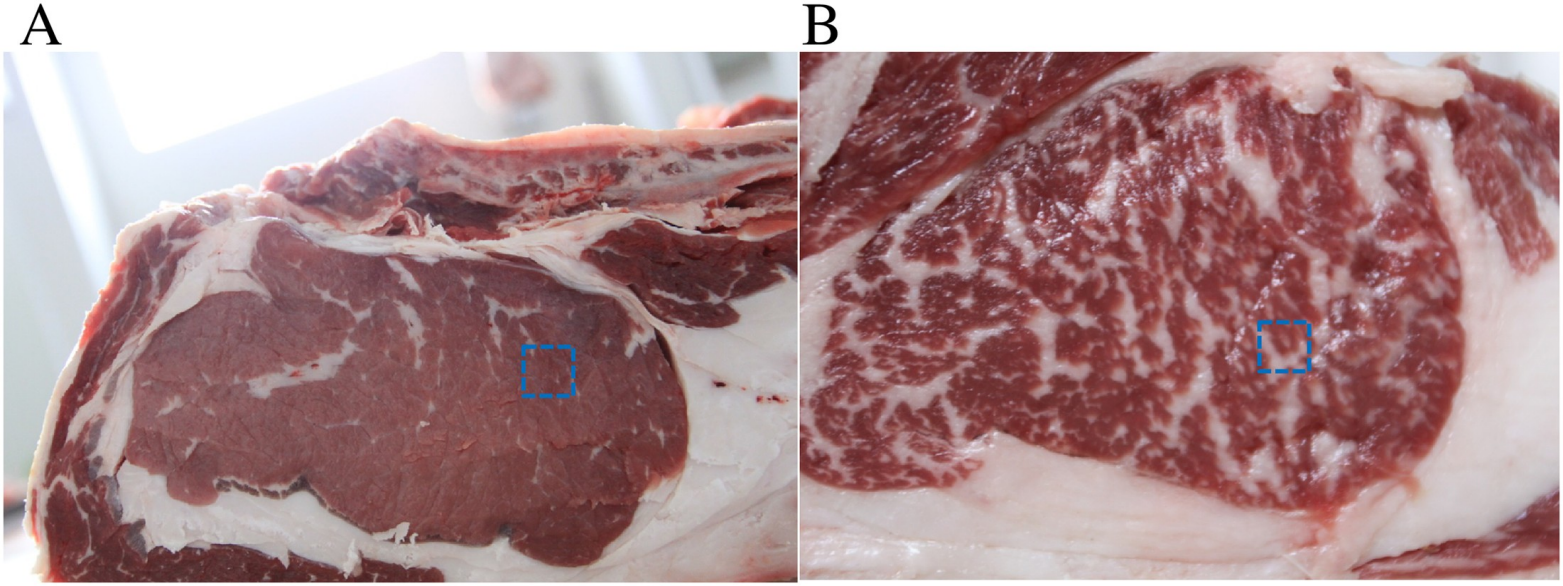
Fat content in the longissimus dorsi in Xinjiang brown cattle. A: Control group A, with the lowest fat content in the longissimus dorsi. B: Group B, with the highest fat content in the longissimus dorsi.

Cattle were slaughtered after being fasted for 12 h. The longissimus dorsi at the 12th and 13th ribs was harvested, labeled and preserved in liquid nitrogen. The fat content in the longissimus dorsi was measured. The two cattle with the lowest fat content in the longissimus dorsi were chosen as control group A, and the two cattle with the highest fat content were chosen as experimental group B. See Fig 1.

### mRNA library construction and sequencing

Total RNA was isolated using a mirVana miRNA Isolation Kit-AM1561 (Ambion, Austin, TX). An enriched cDNA library was constructed using the isolated RNA. Polyacrylamide gel electrophoresis (PAGE) was performed, and the RNA bands were then cut from the gel. Then, 18–30 nt RNA was obtained, and miRNAs were constructed. Finally, miRNA and mRNA sequencing was performed using a BGISEQ-50 sequencing system (BGI).

### Data processing

Impurities were filtered to obtain clean reads. Gene quantification was performed using the RSEM program [10]. DEGs with an FDR ≤0.001 and a fold-change ≥2 were chosen using the Poisson process.

The clean reads obtained by the sRNA sequencing were aligned to reference sequences using HISAT [11] and Bowtie2 [12]. sRNA expression was normalized using TPM [13].

### Prediction of miRNA target genes and correlation analysis between miRNAs and mRNAs

The miRNA target genes were predicted using RNAhybrid, miRanda and TargetScan [14, 15]. The differentially expressed mRNAs and miRNAs were used to construct DEG libraries. Then, a correlation analysis was performed between the DEGs in the two libraries, and differentially expressed target genes that were negatively correlated with miRNAs were selected. An integrated network analysis was conducted. Differentially expressed miRNAs and their target genes were chosen from the different fat-content groups.

### GO and pathway enrichment analyses

GO and pathway enrichment analyses were conducted using ‘GO: TermFinder’ (http://www.yeastgenome.org/help/analyze/go-term-finder). The significantly enriched pathways (Q value ≤0.05) among the DEGs were identified by performing an alignment against the whole genome.

### Construction of miRNA-target interaction network

GO and KEGG pathway analyses were conducted for the DEGs that were negatively correlated with the differentially expressed miRNAs. The genes enriched in both key pathways and GO terms were selected to construct the DE-miRNA network.

### Quantitative validation of the differentially expressed mRNAs and miRNAs

Using GAPDH and U6 as internal reference genes, the relevant differentially expressed mRNAs and miRNAs and their downstream functions were chosen for qRT-PCR validation. The primers were synthesized by Sangon Biotech (Shanghai) Co., Ltd. (S1 Table). The qRT-PCR validation of the mRNAs was performed using ABI Power SYBR Green PCR Master Mix according to the manufacturer’s instructions. The qRT-PCR validation of the miRNAs was performed using a miRcute miRNA qPCR Detection Kit (SYBR Green) according to the manufacturer’s instructions. The qRT-PCR parameters were as follows: 40 cycles of incubation at 37°C for 60 min, 95°C for 10 min, 95°C for 15 s, and 60°C for 1 min. The melting curve was plotted.

## Results

### Analysis of the reads obtained from mRNA and miRNA sequencing

The samples were sequenced using RNA-Seq. This generated 24,136,913–24,137,316 raw reads. After removing low-quality reads, 24,090,787–24,103,791 clean reads were retained. After alignment, the clean reads accounted for 99.80% of the reads in Xinjiang brown cattle.

**Fig 2:**
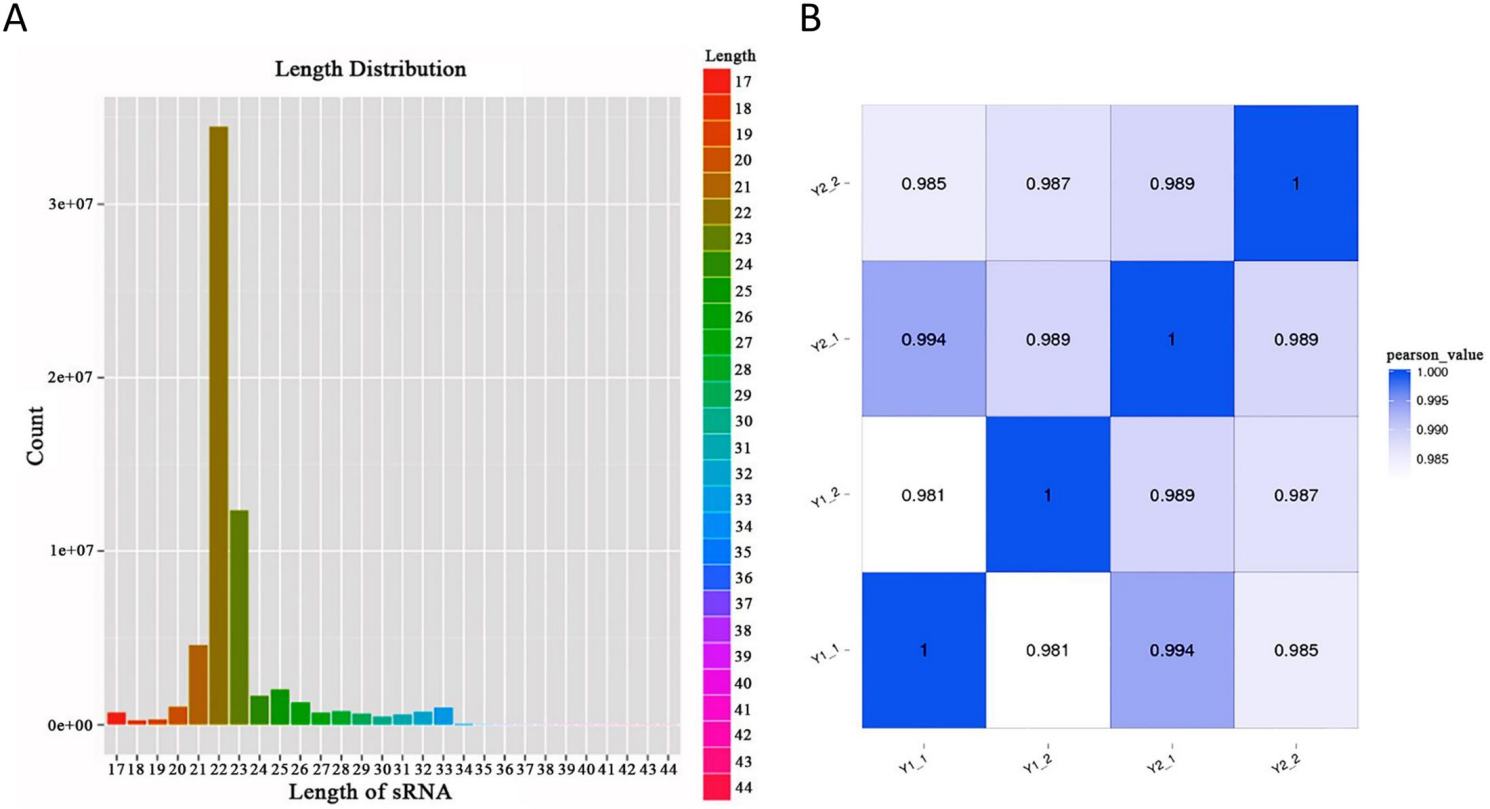
Length distribution of sRNAs and correlations between the samples. A: The x-axis represents the length of the sRNA, and the y-axis represents the proportion of sRNAs with different lengths. The sequence length distribution reflects the types of sRNAs. B: Smaller correlations are closer to white; while larger correlations (closer to 1) are closer to blue.

In total, 24,151,865–71,722,737 raw reads were obtained from 4 sRNA libraries. After filtering out impurities, 22,650,477–64,436,993 clean reads were retained, accounting for 93.84% of the total. The reads were aligned to the known sRNA database to identify sRNAs. Sequences with a length of 18–30 nt accounted for the largest proportion, and this satisfied the requirement for experimental sRNAs, as shown in Fig 2A.

A correlation analysis of the mRNAs among the four samples was then performed, as shown in Fig 2B. The correlation coefficients were all above 0.98, suggesting that the expression patterns were highly similar among the samples.

### Differential mRNA and miRNA expression in longissimus dorsi with different fat contents

**Fig 3:**
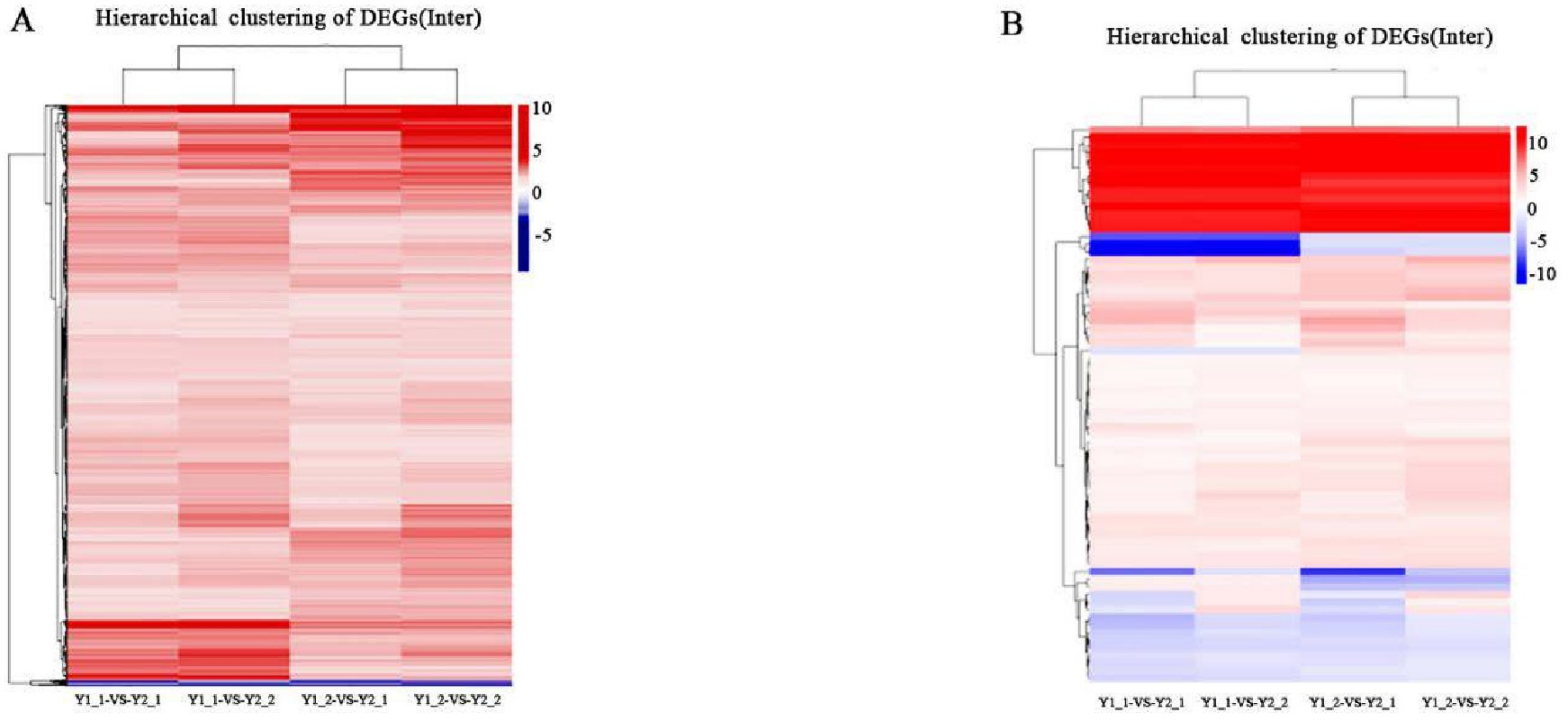
Hierarchical clustering of mRNAs and miRNAs. A: Hierarchical clustering of the DEGs between the groups. B: Hierarchical clustering of the miRNAs. Each row represents a DEG. Different types of DEGs are represented by different colors. Red, upregulated DEGs; blue, downregulated DEGs.

Hierarchical clustering of the DEGs was performed using Cluster software, and the results are shown in Fig 3.

In total, 296 DEGs, including 275 upregulated and 21 downregulated genes, were identified. In all, 362 differentially expressed miRNAs, including 155 newly predicted miRNAs, 252 upregulated genes and 110 downregulated genes, were revealed. The target genes of the differentially expressed miRNAs were predicted, and 209,960 target genes, including 108,256 novel miRNAs, were involved.

In the GO enrichment analysis of the DEGs, 214 of the 296 DEGs were annotated. In all, 712 enriched biological process GO terms, 110 enriched cellular component GO terms, and 111 enriched molecular function GO terms were revealed. Pathway enrichment analysis identified 39 significant pathways, 8 of which were related to lipid metabolism. The enriched lipid metabolism pathways included the biosynthesis of unsaturated fatty acids, fatty acid elongation and fatty acid biosynthesis. After removal of repetitive genes, 11 gene clusters remained. (S2 and S3 Tables).

### GO and pathway enrichment analyses of the target genes of the differentially expressed sRNAs

GO functional analysis was performed on the 22,590 target genes of miRNAs that were differentially expressed between the two groups. In all, 401 cellular component GO terms, 783 molecular function GO terms, and 3,231 biological process GO terms were identified. Pathway enrichment analysis identified 44 significant pathways. The predicted target genes were involved in metabolic pathways, regulation of the actin cytoskeleton, and the MAPK signaling pathway. There were 14 significant pathways. The enriched lipid metabolism pathways included fatty acid degradation, ether lipid metabolism, and glycerophospholipid metabolism. After removal of repetitive target genes, 460 clusters remained, as shown in S3 Table.

### Correlation analysis of the target genes of the differentially expressed miRNAs and differentially expressed mRNAs

**Fig 4:**
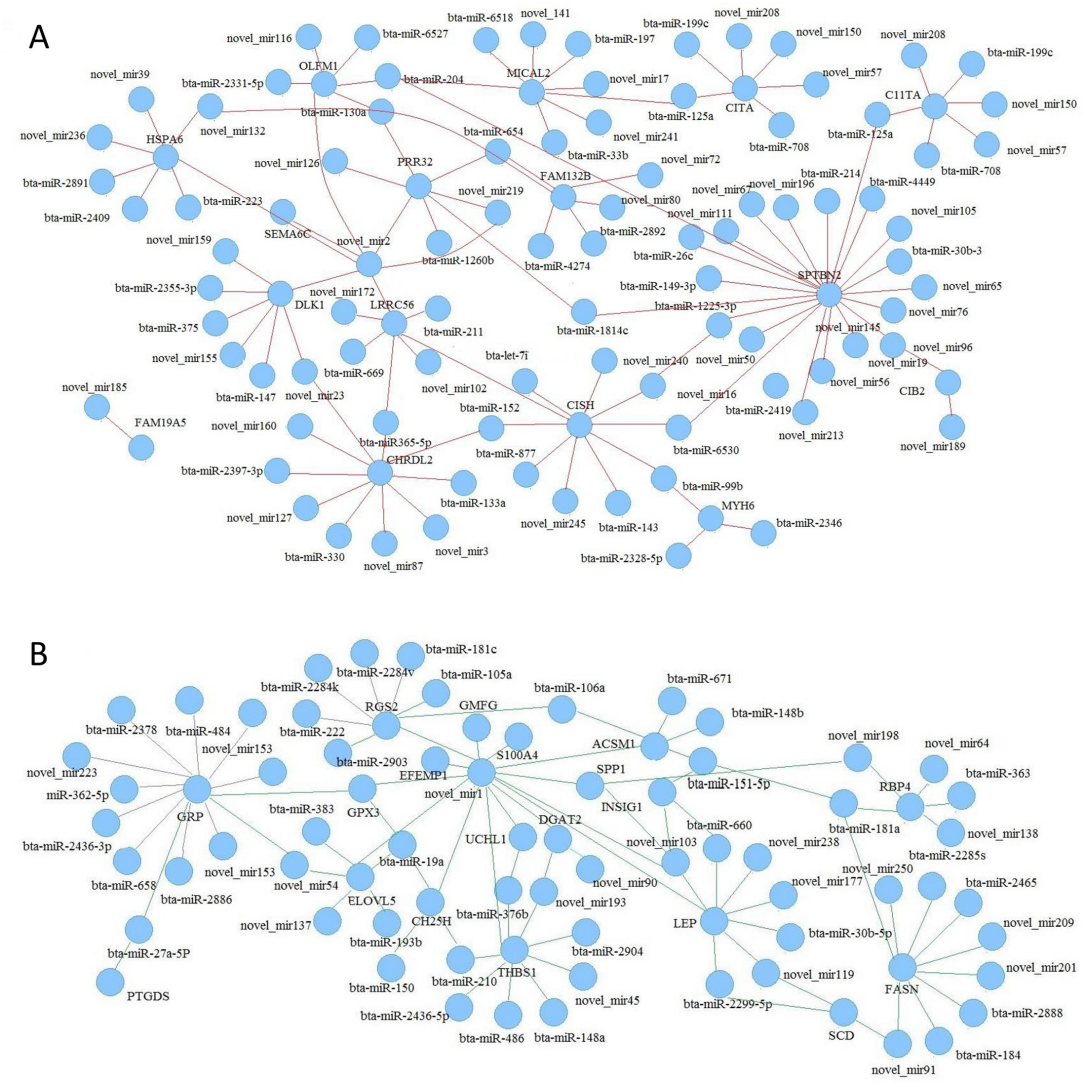
miRNA-target interaction network. A: Upregulated miRNAs and their downregulated target genes. B: Downregulated miRNAs and their upregulated target genes. Red lines represent the intensity of the interaction between the upregulated miRNAs and their downregulated target genes; green lines represent the intensity of the interaction between the downregulated miRNAs and their upregulated target genes.

DEG and target gene libraries were constructed considering that miRNAs post-transcriptionally regulate, degrade or inhibit translation [16]. A correlation analysis of the genes from the two libraries was conducted. DEGs that were negatively correlated with the miRNAs and were related to lipid metabolism were chosen, and a regulatory network was constructed. In all, 96 differentially expressed miRNAs in groups A and B were correlated to 27 target genes. There were 47 upregulated miRNAs and 49 downregulated miRNAs. The 47 upregulated miRNAs had 14 downregulated target genes, and the 49 downregulated miRNAs had 13 upregulated target genes. According to the miRNA-target network (Fig 4), the miRNAs novel-mir2, novel-mir240, miR-130a, miR-125a, miR-181a and miR-30b-5p were located at the center.

### GO and pathway enrichment analyses of the differentially expressed target genes of the miRNAs

GO enrichment analysis revealed that the differentially targeted genes belonged to 71 biological processes, including fatty acid metabolic process and neutral lipid metabolic process; 41 cellular components, including extracellular region part, extracellular region, and cytoplasmic region; and 33 molecular functions, including fatty acid synthase activity, lipase inhibitory activity, and lipid binding. Pathway enrichment analysis showed that the upregulated DEGs belonged to 16 pathways, including biosynthesis of unsaturated fatty acids, fatty acid metabolism, and fatty acid elongation, and the downregulated DEGs belonged to 8 pathways, including the MAPK signaling pathway, glutamatergic synapses, and the glucagon signaling pathway (S4 Table).

### qRT-PCR validation of the differentially expressed miRNAs and mRNAs

**Fig 5:**
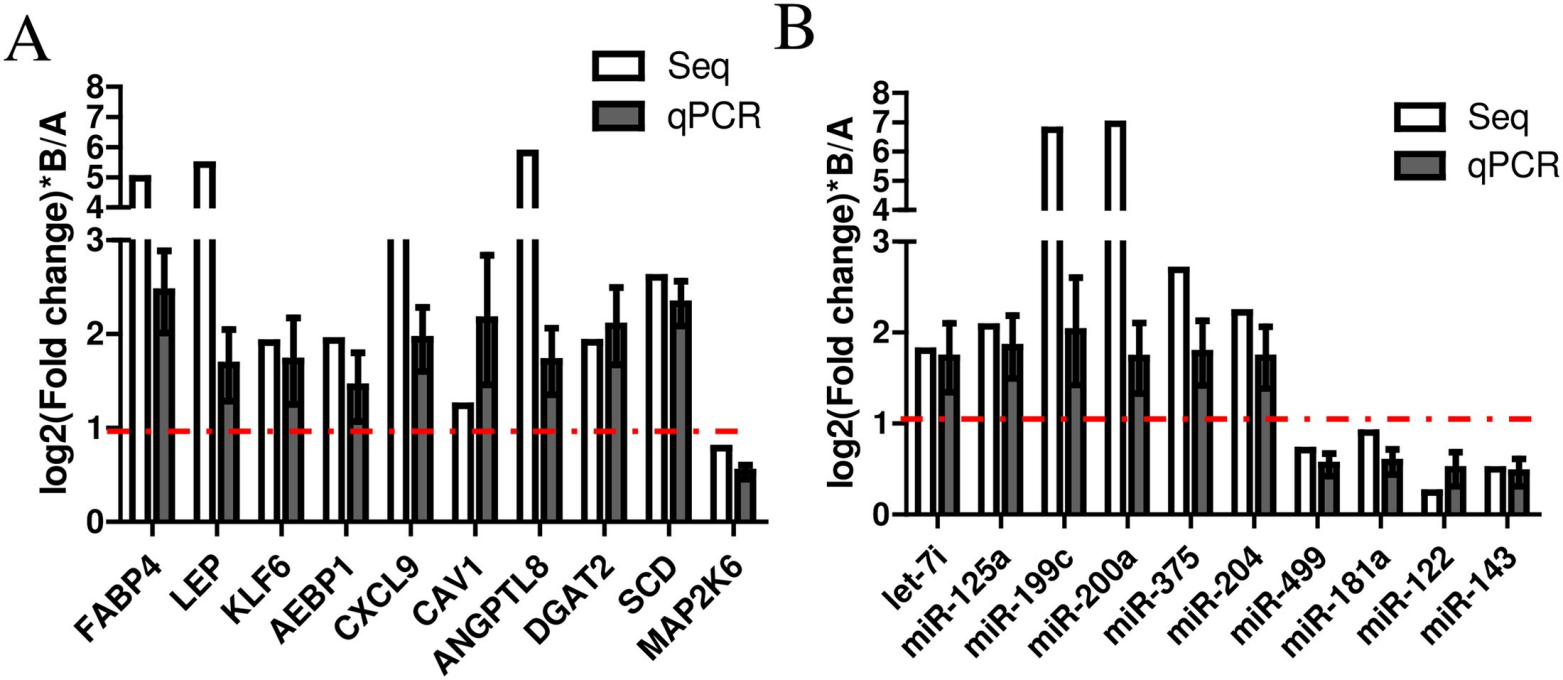
qRT-PCR validation of the differentially expressed miRNAs and mRNAs. A: Comparison of the mRNA sequencing and qRT-PCR results. B: Comparison of the miRNA sequencing and qRT-PCR results.

Based on the combined analysis of the miRNAs and mRNAs, we selected 10 DEGs for fluorescent quantitative PCR. FABP4, LEP, KLF6, AEBP1, CXCL9, CAV1, ANGPTL8, DGAT2, SCD and MAP2K6 were associated with adipogenesis and adipocyte differentiation. Let-7i-5p, miR-125a, miR-199a-5p, miR-200b, miR-143, miR-122, miR-375, miR-204-5p, miR-181a and miR-429 affect adipocyte differentiation. The qRT-PCR validation confirmed that the differentially expressed miRNAs and mRNAs obtained by sequencing were accurate, as shown in Fig 5.

## Discussion

Meat quality is determined on the basis of marbling, texture, fat color, meat color and overall maturity [17]. Fat deposition is positively related to beef flavor and palatability. The deposition of intramuscular adipose tissue influences both meat quality and animal productivity [18]. Understanding the molecular regulation of adipose tissue is a key step toward improving beef quality. Bovine adipose tissue may be regulated by upstream mechanisms such as miRNAs [19] and transcriptional factors [20]. However, few studies have investigated the differential expression of miRNAs related to intramuscular fat content in the longissimus dorsi in Xinjiang brown cattle. Garin et al. (2014) found that FABP4, also known as aP2, suppresses PPARγ and adipogenesis [21]. Bojlul et al. (2013) showed that leptin plays an important role in energy homeostasis [22]. Lee et al. (2012) found that KLF8 is an upstream regulator of PPARγ and C/EBPα and is a key component during adipogenesis [23]. Boqué et al. (2013) revealed that apple polyphenol (AP) intake led to decreased adipocyte enhancer-binding protein (AEBP1) mRNA levels in epididymal adipocytes [24]. Gerst et al. (2017) showed that palmitate and fetuin-A induced CXCL8 mRNA expression in differentiated adipocytes [25]. Sara et al. (2015) found that Caveolin-1 (Cav-1) is a key component of adipocyte caveolae and plays an important role in regulating insulin signaling [26]. Bruno et al. (2016) revealed that Angiopoietin-like protein 8 (ANGPTL8) is implicated in lipid and glucose homeostasis [27]. Ryo et al. (2005) showed that diacylglycerol acyltransferase (DGAT) enzymes facilitated the final step in mammalian triglyceride synthesis and that DGAT was associated with obesity, insulin resistance, and leptin resistance [28]. Niclas et al. (2011) found that Stearoyl-CoA desaturase-1 (SCD-1) is a key enzyme in lipogenesis and that inhibiting its activity impairs lipid synthesis [29]. Jihye et al. (2012) revealed that MAP2K6 affects carcass weight (CW), back fat thickness (BFT) and marbling score (MS) [30].

Giroud et al. (2016) showed that Let-7i-5p was downregulated and repressed brite adipocyte function in human brite adipocytes [31]. Ji et al. (2014) revealed that miR-125a suppresses porcine preadipocytes differentiation by downregulating ERRa [32]. Shi et al. (2014) found that miR-199a-5p facilitated cell proliferation and inhibited lipid deposition in porcine adipocytes by directly targeting Cav-1 [33]. Shen et al. (2018) reported that miR-200b inhibited preadipocyte proliferation by attenuating KLF4 [34]. Kim et al. (2015) showed that miR-143 facilitated adipogenesis by directly targeting pref-1 in adipocytes [35]. Liao et al. (2018) revealed that miR-122 regulated PPAR-γ signaling and adipocyte differentiation in vitro and in human adipose tissue [36]. Liu et al. (2016) found that miR-375 inhibited porcine preadipocyte differentiation by directly targeting BMPR2 [37]. Du et al. (2018) reported that miR-204-5p promoted adipocyte differentiation by directly targeting KLF3 [38]. Li et al. (2013) found that miR-181a accelerated lipid droplet accumulation and increased triglyceride levels by negatively regulating TNF-α [39]. Jiang et al. (2018) showed that miR-499 represses adipogenic differentiation by directly targeting PRDM16 in skeletal muscle satellite cells (SMSCs) [40].

Along with the increased depth provided by next-generation sequencing, RNA-Seq has become a powerful tool for performing comprehensive transcriptome analyses [41]. Compared with microarray and SAGE technologies, RNA-Seq is more sensitive and more widely used [42]. The massive data obtained from RNA-Seq can be analyzed using Bowtie2 and TargetScan software [15]. Here, we applied RNA-Seq to sequence the longissimus dorsi in Xinjiang brown cattle, and the data were analyzed using Bowtie2 software. The lean reads obtained from the transcriptome library accounted for over 95% of the original reads. Moreover, the clean reads obtained from the sRNA library accounted for over 93%, indicating that most known miRNAs are expressed in the muscles of Xinjiang brown cattle. However, not all miRNAs were detected, indicating the tissue specificity of the miRNAs [43]. From another perspective, more tissue-specific miRNAs will likely be discovered in cattle in the future.

Establishing biological replicates is required in any biological experiment, and high-throughput sequencing is no exception [44]. Correlating gene expression between different samples is an important indicator of experimental reliability and the reasonability of sample selection. The closer the correlation coefficient is to 1, the higher the similarity between the two samples. We used only two biological replicates in this study, and this may have led to false-positive results. According to FPKM, the correlation coefficients between the two groups were all above 0.985, which satisfied the experimental requirement. Furthermore, 10 miRNAs and 10 miRNAs were selected from 6 samples for qRT-PCR validation, and the qRT-PCR results were consistent with the sequencing results, supporting the reliability of the DEGs obtained by RNA-Seq. Thus, using only one or two biological replicates was sufficient to identify key DEGs.

miRNAs are widely present in the genomes of animals and plants and play regulatory roles in lipid metabolism in cattle. Jin detected miRNAs in adipose tissues on the backs of three types of crossbred cattle with different thicknesses of back fat. Eighty-nine miRNAs were detected, including 42 differentially expressed miRNAs, with the expression of miR-378 being the most significantly different [45]. This finding was confirmed in our study. Li observed that miR-143 was upregulated in intramuscular adipocytes that differentiated into mature adipocytes in cattle [46]. Once the expression of miR-143 was inhibited, the differentiation of the precursors of the intramuscular adipocytes was also inhibited. In the present study, miR-143 expression was significantly higher in the experimental group than in the control group, suggesting that miR-143 plays a potential role in the formation of intramuscular fat in cattle.

To further explore the potential regulatory role of the aforementioned miRNAs, the target genes of the differentially expressed miRNAs were predicted. In all, 209,960 target genes were involved, and the miRNA-target interaction network indicated that one miRNA may regulate several dozens or hundreds of target genes. In addition, each target gene was regulated by several miRNAs. In this study, 1,074 target genes were predicted for miR-143, which exhibited the largest fold-change difference between the two groups.

The post-transcriptional regulation of target genes by miRNAs has been proved in numerous studies [47–49]. Here, RNA-Seq was innovatively applied to perform a combined analysis of the target genes of differentially expressed miRNAs and differentially expressed mRNAs. Ninety-six negatively correlated miRNAs and 27 target genes were identified. These 27 target genes are key genes responsible for the variability in the fat content and meat quality traits of the longissimus dorsi in Xinjiang brown cattle. The related pathways were the focus of this study. These differentially expressed target genes belonged to 71 biological processes, 41 cellular components and 33 molecular functions. Pathway enrichment analysis indicated that the upregulated DEGs belonged to 16 pathways, including the biosynthesis of unsaturated fatty acids, fatty acid metabolism, the pentose phosphate pathway and fatty acid elongation, while the downregulated DEGs belonged to 8 pathways, including the MAPK signaling pathway, glutamatergic synapses and hypertrophic cardiomyopathy (HCM). The MAPK signaling pathway, which was the most significantly enriched pathway, is involved in the conversion of extracellular signals into intracellular signals in eukaryotes. Studies have shown that miRNAs can target certain genes in the MAPK signaling pathway, thus influencing signal transduction and participating in adipocyte metabolism [50–52]. Lin et al. found that PHB was a target gene of miR-27a and miR-27b [53]. PHB is highly expressed in adipocytes and is closely associated with adipocyte differentiation. PHB overexpression in 3T3-L1 cells inhibits insulin-induced adipogenic differentiation. However, many pathways may influence adipocyte differentiation, and MAPK signaling is but one such key pathway. Moreover, the pRB-E2F, Wnt, biosynthesis of unsaturated fatty acids and fatty acid metabolism pathways identified in this study also play a role in adipocyte metabolism. These pathways, together with miRNAs, form a large network that regulates cell differentiation.

## Conclusion

We applied RNA-Seq to the longissimus dorsi of Xinjiang brown cattle and identified 24,098,031 clean reads. The average alignment rate of the clean reads against the reference genome was 95.35%. sRNA sequencing produced 46,007,468 clean reads, which accounted for 93.33% of the total reads. Thus, the sequencing had a high yield and effectively detected mRNAs and miRNAs in the tissues.

Four mRNA and sRNA libraries were constructed for the longissimus dorsi of Xinjiang brown cattle, and 296 differentially expressed mRNAs and 362 differentially expressed miRNAs were identified as follows: 155 newly predicted miRNAs, 275 upregulated genes, 252 upregulated miRNAs, 21 downregulated genes and 110 downregulated miRNAs. The combined analysis of the miRNAs and mRNAs revealed 96 differentially expressed miRNAs and 27 differentially expressed mRNAs. We found that 47 upregulated miRNAs had a regulatory effect on 14 downregulated target genes and that 49 downregulated miRNAs had a regulatory effect on 13 upregulated target genes. An miRNA-target interaction network was constructed. Of the DEGs, those in metabolic pathways, especially pathways related to lipid metabolism, were enriched by the largest proportions.

## Supporting information

S1 Table Primers for reverse transcription and quantitative real-time PCR. (DOCX)

S2 Table Differential expression of miRNAs in longissimus dorsi with different fat content. (XLS)

S3 Table Differential expression of mRNAs in longissimus dorsi with different fat content. (XLS)

S4 Table Differentially expressed target genes of miRNAs. (XLS)

S5 Table Raw data. (XLS)
