# Supplementary material for: Differential expression of mRNA-miRNAs related to intramuscular fat content in the longissimus dorsi in Xinjiang brown cattle
Source: PLoS One. 2018 Nov 9;13(11):e0206757. doi: 10.1371/journal.pone.0206757 (PMC6226300; doi:10.1371/journal.pone.0206757)
Supplement: S1 Table — (DOCX) [file pone.0206757.s001.docx]

S5 Table. Primers for reverse transcription and quantitative real-time PCR.

| Primers | Sequences (From 5′to 3′) |
| --- | --- |
| FABP4-F | TGTCACTGCCACCAGAGTTT |
| FABP4-R | TGGACAACGTATCCAGCAGA |
| LEP-F | CCCTGTATCGATTCCTGTGGC |
| LEP-R | GACGGACTGCGTGTGTGAGAT |
| KLF6-F | CTAATCGCTGCGCACTTGAG |
| KLF6-R | GTCCCCGCTGCTGTAAAAAC |
| AEBP1-F | AGTTCCTCGAGGGCTTCCTGT |
| AEBP1-R | GTCCTTCTTCCCTTTCTTCCCTT |
| CAV1-F | GTCCTTCTTCCCTTTCTTCCCTT |
| CAV1-R | GGGGGCAAATACGTAGACTCA |
| ANGPTL8-F | AGTGAGCATTGATCCAGGGC |
| ANGPTL8-R | CAGTAGTTCACCCGGAGCTG |
| DGAT2-F | GGGTCCTGTCTTTCCTCGTG |
| DGAT2-R | CAGGGCCAGTTTCACAAAGC |
| CXCL9-F | ATGGACGCTGTTCCTGCATC |
| CXCL9-R | TGGGTTTAGGCAGGCTTCATT |
| SCD-F | TGGGTTTAGGCAGGCTTCATT |
| SCD-R | TCCGACCTAAGAGCCGAGAA |
| MAP2K6-F | ACGTTGCAACTGGGGGAAAA |
| MAP2K6-R | CGAGGCAGCAGATGGACTTC |
| GAPDH-F | CACCCTCAAGATTGTCAGCA |
| GAPDH-R | GGTCATAAGTCCCTCCACGA |
| U6-RT | CGCTTCACGAATTTGCGTGTCAT |
| U6-F | GCTTCGGCAGCACATATACTAAAAT |
| U6-R | CGCTTCACGAATTTGCGTGTCAT |
| Let-7i-RT | CTCAACTGGTGTCGTGGAGTCGGCAATTCAGTTGAGAACAGCAC |
| Let-7i -F | ACACTCCAGCTGGGTGAGGTAGTAGTTTGT |
| MiR-125a-RT | CTCAACTGGTGTCGTGGAGTCGGCAATTCAGTTGAGCACAGGTT |
| MiR-125a -F | ACACTCCAGCTGGGTCCCTGAGACCCTTTAA |
| MiR-199c-RT | CTCAACTGGTGTCGTGGAGTCGGCAATTCAGTTGAGCCAATGTG |
| MiR-199c -F | ACACTCCAGCTGGGTACAGTAGTCTGCA |
| MiR-200a-RT | CTCAACTGGTGTCGTGGAGTCGGCAATTCAGTTGAGAACATCGT |
| MiR-200a-F | ACACTCCAGCTGGGTAACACTGTCTGGTAAC |
| MiR-375-RT | CTCAACTGGTGTCGTGGAGTCGGCAATTCAGTTGAGTCACGCGA |
| MiR-375-F | ACACTCCAGCTGGGTTTTGTTCGTTCGGCTC |
| MiR-204-RT | CTCAACTGGTGTCGTGGAGTCGGCAATTCAGTTGAGAGGCATAG |
| MiR-204 -F | ACACTCCAGCTGGGTTCCCTTTGTCATCCT |
| MiR-499-RT | CTCAACTGGTGTCGTGGAGTCGGCAATTCAGTTGAGAAACATCA |
| MiR-499 -F | ACACTCCAGCTGGGTTAAGACTTGCAGTG |
| MiR-181a-RT | CTCAACTGGTGTCGTGGAGTCGGCAATTCAGTTGAGAACTCACC |
| MiR-181a-F | ACACTCCAGCTGGGAACATTCAACGCTGTCGG |
| MiR-122-RT | CTCAACTGGTGTCGTGGAGTCGGCAATTCAGTTGAGCAAACACC |
| MiR-122-F | ACACTCCAGCTGGGTGGAGTGTGACAATGG |
| MiR-143-RT | CTCAACTGGTGTCGTGGAGTCGGCAATTCAGTTGAGCGAGCTAC |
| MiR-143 -F | ACACTCCAGCTGGGTGAGATGAAGCACTGT |
| Universal reverse | CTCAAGTGTCGTGGAGTCGGCAA |
